# Effects of Asiatic Acid on Spatial Working Memory and Cell Proliferation in the Adult Rat Hippocampus

**DOI:** 10.3390/nu7105401

**Published:** 2015-10-05

**Authors:** Apiwat Sirichoat, Wunnee Chaijaroonkhanarak, Parichat Prachaney, Wanassanan Pannangrong, Ratana Leksomboon, Amnart Chaichun, Peter Wigmore, Jariya Umka Welbat

**Affiliations:** 1 Department of Anatomy, Faculty of Medicine, Khon Kaen University, Khon Kaen 40002, Thailand; dek_saba@hotmail.com (A.S.); cwunnee@kku.ac.th (W.C.); parpra@kku.ac.th (P.P.); wankun@kku.ac.th (W.P.); anmcha@kku.ac.th (A.C.); 2 Center for Research and Development of Herbal Health Products, Khon Kaen University, Khon Kaen 40002, Thailand; 3 College of Medicine and Public Health Ubon Ratchathani University, Ubon Ratchathani 34190, Thailand; ratana_tlek@yahoo.com; 4 School of Life Sciences, Medical School, Queen’s Medical Centre, Nottingham University, Nottingham NG7 2UH, UK; peter.wigmore@nottingham.ac.uk; 5 Neuroscience Research and Development Group, Khon Kaen University, Khon Kaen 40002, Thailand

**Keywords:** hippocampus, cell proliferation, spatial memory.

## Abstract

Asiatic acid is a pentacyclic triterpene from *Centella asiatica*. Previous studies have reported that asiatic acid exhibits antioxidant and neuroprotective activities in cell culture. It also prevents memory deficits in animal models. The objective of this study was to investigate the relationship between spatial working memory and changes in cell proliferation within the hippocampus after administration of asiatic acid to male Spraque-Dawley rats. Control rats received vehicle (propylene glycol) while treated rats received asiatic acid (30 mg/kg) orally for 14 or 28 days. Spatial memory was determined using the novel object location (NOL) test. In animals administered asiatic acid for both 14 and 28 days, the number of Ki-67 positive cells in the subgranular zone of the dentate gyrus was significantly higher than in control animals. This was associated with a significant increase in their ability to discriminate between novel and familiar object locations in a novel object discrimination task, a hippocampus-dependent spatial memory test. Administration of asiatic acid also significantly increased doublecortin (DCX) and Notch1 protein levels in the hippocampus. These findings demonstrate that asiatic acid treatment may be a potent cognitive enhancer which improves hippocampal-dependent spatial memory, likely by increasing hippocampal neurogenesis.

## 1. Introduction

Asiatic acid, one of the major components of *Centella asiatica* (L.) Urban [1], is a tropical medicinal plant from the Apiaceae family local to Southeast Asian countries including India, China, Sri Lanka, Malaysia and Thailand [2,3,4]. *Centella asiatica* is regarded as an essential herb in Ayuveda and is believed to improve learning and memory [5]. Asiatic acid, an active component of *C. asiatica*, is a triterpenoid, with reported neuroprotective activity both *in vitro* and *in vivo* [6,7,8]. In cell culture, asiatic acid prevents C_2_-ceramide induced cell death in primary cortical neurons, an action brought about by stimulation of cellular oxidative defense pathways [4]. *In vivo*, 30 mg/kg of asiatic acid significantly improves learning and memory in rats through modulation of the cholinergic and GABAergic systems [3]. It has also been found to reduce infarct volume in animal models of stroke [8]. One mechanism by which asiatic acid may be exerting its effect is by acting as an anti-oxidant and reducing levels of reactive oxygen species.

New neurons are generated from dividing neural stem cells (NPCs) throughout life in a small number of adult brain regions [9,10]. The subventricular zone (SVZ) of the lateral ventricle and the subgranular zone (SGZ) of the hippocampal dentate gyrus are the two main neurogenic regions in the brain [11,12]. Stem cell proliferation in the SGZ generates granule cell neurons which integrate into the circuitry of the dentate gyrus and contributes to hippocampal function. The rate of neurogenesis has been correlated with the ability to perform hippocampal-dependent learning and memory tasks [13]. The hippocampal dentate gyrus is strongly activated during the initial stages of spatial learning in response to novel spatial conditions [14]. Moreover, newborn neurons in the dentate gyrus promote exploratory behavior, increase synaptic plasticity in the hippocampus, enable the rapid acquisition of information for spatial memory formation and are preferentially activated during the process of learning [15,16].

No studies have examined the impact of asiatic acid on hippocampal neurogenesis or on spatial working memory. The present study aims to investigate the effect of this compound on spatial working memory using the novel object location (NOL) behavioral test. This test requires an intact dentate gyrus and has been shown to be sensitive to levels of hippocampal neurogenesis [17,18,19,20]. As other drugs such as antidepressants are only effective after chronic (>3 weeks) but not shorter treatment periods [21], asiatic acid was administered for 14 or 28 days. As Ki-67 protein is expressed during all active phases of the cell cycle (G_1_, S_1_, G_2_ and mitosis) but not in the resting phase (G_0_) [22], Ki-67 was used to quantify the numbers of dividing cells in the SGZ of the dentate gyrus at the end of the experiment. Finally, Western blotting was used to quantify the levels of Notch1, a receptor expressed by neural stem cells and doublecortin (DCX), a newborn neuron marker, in the hippocampus.

## 2. Materials and Methods

### 2.1. Animals and Treatments

For this study, 4-week old male Spraque-Dawley rats (National Laboratory Animal Center, Mahidol University, Bangkok, Thailand) weighing 180–200 grams were used for all experiments. The experimental protocol was approved by the Khon Kaen University Ethics Committee in Animal Research (project number. AEKKU 30/2556). Rats were group-housed in a 12 h light/dark cycle with ad libitum food and water. After 7 days of habituation, animals were randomly allocated to control (*n* = 20) and drug-treated (*n* = 20) groups. Control rats were orally administered propylene glycol (1 mL/kg, Ajax Finechem Pty Ltd., Auckland, New Zealand) for 14 or 28 days while drug-treated rats orally received asiatic acid (30 mg/kg/day, dissolved in propylene glycol, Faces Biochemical Co., Ltd., Wuhan, China) via gavage tube in a volume of 1 mL/kg for 14 or 28 days.

### 2.2. Behavioral Testing

The novel object location test was used to determine spatial working memory after the drug administration. The protocol was modified from the original method [23] and carried out as described previously [20,24,25]. The apparatus consisted of an arena (a semi-transparent plastic box, dimensions 36-cm wide × 50-cm long × 36-cm high) and plastic bottles filled with water to weigh them down. Experiments were conducted at an illumination of 350–400 lux and recorded by VDO camcorder Version-052, OKER, Crown computer Co., Ltd, Bangkok, Thailand).

The NOL test was performed 3 days after the end of 14 and 28 days of drug treatment. Animals were habituated by allowing them to freely explore an open-field arena in the absence of objects for 30 min, one day prior to testing (10 animals per group). The task procedure comprised a familiarization and a choice trial with a 5 min inter-trial interval. In the familiarization trial, two identical objects were placed in separate locations in the arena and each animal was allowed to explore the objects for 3 min. Then, the animals were returned to their home cages. Meanwhile the objects and the arenas were cleaned with 20% ethanol to eliminate olfactory cues. During the choice trial, the animals were returned to the arena for 3 min, in which one object remained in the same familiar location while the other object had been moved to a new (novel) location.

A positive exploration of the objects was scored when the animal directed its nose at a distance less than 2 cm from the object [23]. Exploratory time was scored as total amount of time spent on each object (familiar and novel locations). The preference index was defined as time spent exploring the object in the novel location in the choice trial as a percentage when compared to 50% chance [23].

### 2.3. Tissue Preparation

The day after the NOL test, rats were put down by rapid stunning followed by decapitation [20]. Ten brains from each group were removed and divided; half of each brain was cryoprotected in a 30% sucrose solution for 3 h at 4 °C and then embedded in Optimal Cutting Temperature (OCT) compound (Sakura Finetek USA, Torrance, CA, USA). These were snap-frozen in liquid nitrogen–cooled isopentane and stored at −80 °C prior to sectioning. The hippocampai were dissected out from the other brain halves and snap-frozen in liquid nitrogen and stored at −80 °C for subsequent Western immunoblotting.

### 2.4. Immunohistochemistry

6 randomly selected frozen brains were serially sectioned (20 µm) in the coronal plane from the Bregma point −2.3 to −6.3 mm to include the entire dentate gyrus using a cryostat. Sections were mounted on 3-aminopropyl-methoxysilane (APS) coated slides and stored at −20 °C. A systematic random sampling method [26] was used to choose every 15th section throughout the length of the dentate gyrus (9 sections from each brain).

Ki-67 staining was carried out as described previously [20]. Sections were fixed in 0.5% paraformaldehyde (pH 7.4) for 3 min and then incubated with monoclonal mouse Ki-67 (1:150, Vector Laboratory, Inc., Burlingame, CA, USA) at room temperature for 1 h. Following washing, sections were incubated with Alexa 488 rabbit anti-mouse IgG (1:300, Invitrogen, Eugene, OR, USA) for 40 min and counter-stained with propidium iodide (1:6000, Sigma Aldrich, Inc., St. Louis, MO, USA) for 30 s and mounted in glycerol.

All sections were viewed and quantified at X40 on a Nikon ECLIPSE 80i fluorescence microscope with NIS-Element AR 3.2 software (Melville, NY, USA). Ki-67 positive cells which were co-localized with propidium iodide nuclear staining and were within 3 cell diameters of the inner edge of both blades of the dentate gyrus were scored [27]. The number of Ki-67 positive cells in each hippocampus was produced by combining cell counts per section for the whole dentate gyrus and multiplying by 15 [28].

### 2.5. Immunoblotting

Sodium Dodecyl Sulfate-Polyacrylamide Gel Electrophoresis (SDS-page) and Western blots were performed using standard protocols [20]. 30 μg of protein per lane was loaded onto 12% SDS-polyacrylamide gels to detect DCX, while 50 μg proteins per lane were loaded onto 10% SDS-gels to quantify Notch1 levels. Proteins were transferred onto nitrocellulose membranes. Blots were incubated with primary antibodies, polyclonal anti-DCX (1:150, Santa Cruz, CA, USA), polyclonal anti-Notch1 (1:100, Santa Cruz, CA, USA) and monoclonal mouse anti-GAPDH antibody (1:20,000 Abcam, Cambridge, UK) over night at 4 °C. The blots were washed and incubated with secondary antibody (1:2000, polyclonal goat anti-mouse and polyclonal rabbit anti-goat, Santa Cruz, CA, USA). Then, blots were exposed to a chemiluminescent detection system using an ECL solution (GE Healthcare, Buckinghamshire, UK). The images were quantified using densitometry measurement using ImageJ software (version 1.48q, Rayne Rasband,National Institutes of health, USA). Levels of Notch1 and DCX were normalized to Glyceraldehyde 3-phosphate dehydrogenase (GAPDH) and data were presented as DCX and Notch1 optical density.

### 2.6. Statistical Analysis

All statistical parameters were calculated using GraphPad Prism (V. 5.0), Statistical Package for Social Sciences (SPSS) (V 17.0) (SPSS Inc.,Chicago, USA) and expressed as mean ± standard error of mean (SEM). A probability level of *p* < 0.05 was considered statistically significant. The Student *t-*test and ANOVA were used to analyze data. Least Significant Difference (LSD) *post hoc* test was performed when Analysis of variance (ANOVA) was significant.

## 3. Results

### 3.1. Effect of Asiatic Acid on Spatial Working Memory

**Figure 1:**
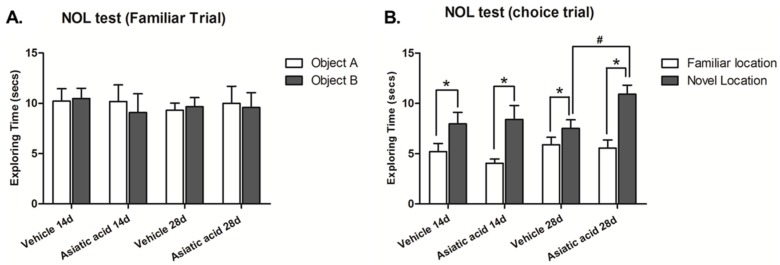
Mean exploration times of the animals exploring each object during the familiarization (**A**) and choice (**B**) trials of the novel object location test after treatment. There were no significant differences in exploration times of either object for any group in the familiarization (10 animals per group). In the choice trial, all groups spent a significantly longer time exploring the object in the novel location when compared with the familiar location (*p* < 0.05). Moreover, exploration times of the novel location in animals that received asiatic acid for 28 days was significant higher than 14 days (*p* < 0.05). Statistical assessment was by paired Student *t*-test. NOL: Novel Object Location.

Spatial working memory was assessed using the NOL test. The animals in all groups showed no significant difference in exploratory time for each object in the familiarization trial (Figure 1A), suggesting that there was no preference for either object’s location prior to the choice trial. During the choice trial, one object was moved to a new location, the animals in all groups spent significantly more time attending the object in the novel location (mean ± SEM); control 14 days-treated group: 7.972 ± 1.137 s, asiatic acid 14 days-treated group: 8.408 ± 1.384 s, control 28 days-treated group: 7.519 ± 0.850 s, asiatic acid 28 days-treated group: 10.920 ± 0.907 s; *n* = 10; *p* < 0.05, paired Student *t*-test, Figure 1B). These results indicate that the animals preferred the object in the novel location over that in the familiar location (mean ± SEM; control 14 days-treated group: 5.201 ± 0.823 s, asiatic acid 14 days-treated group: 4.052 ± 0.417 s, control 28 days-treated group: 5.894 ± 0.743 s, asiatic acid 28 days-treated group: 5.564 ± 0.807 s). An Fisher's LSD *post hoc* test confirmed that animals that received asiatic acid for 28 days spent significantly more time on the objects located in the novel locations in comparison to control animals (F(1, 36) = 3.092; *n* = 10; *p* < 0.05).

**Figure 2:**
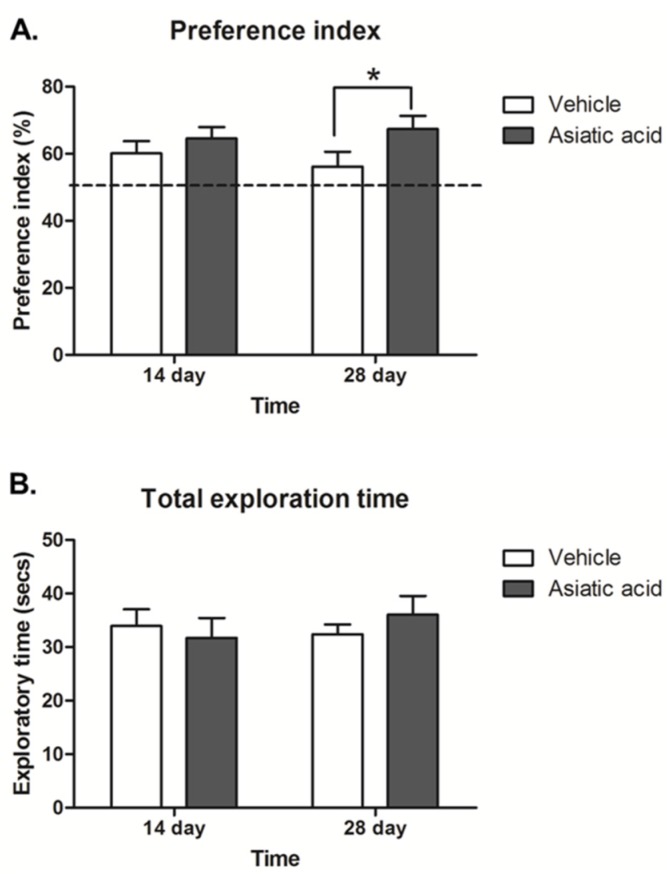
The preference index (PI) showed a significant difference from 50% chance in all groups (**A**; *p* < 0.05). The animals received asiatic acid for 28 days, but they had a significantly higher PI compared to controls (* *p* < 0.05); The total exploratory time (**B**) in familiarization and choice trials in all groups was not significantly different; a two-way Analysis of variance with Fisher's Least Significant Difference *post hoc* test was used to compare between all groups.

The preference index (PI) was calculated by expressing time spent exploring the objects in the novel locations in the choice trial as a percentage compared to 50% chance. The data showed that all animals had a significantly higher PI than a 50% chance (*p* < 0.05; *n* = 10; one-sample *t*-test, Figure 2A), indicating a normal ability to remember the location of object and express greater interest in objects in novel locations. The PI in animals that had received asiatic acid for 28 days but not 14 days was significantly higher than the control group (F (1,36) = 4.062; *n* = 10; *p* < 0.05, two-way ANOVA, LSD *post-hoc* test, Figure 2A). These findings indicate that animals that received asiatic acid for 28 days showed enhanced spatial discrimination compared to controls.

The total exploration times showed no significant differences among groups, indicating that animals did not have impaired locomotor ability during the performance of the task (F (1,36) = 0.054; *n* = 10; *p* > 0.05, two-way ANOVA, LSD *post-hoc* test, Figure 2B).

### 3.2. Effect of Asiatic Acid on Cell Proliferation in the SGZ

**Figure 3:**
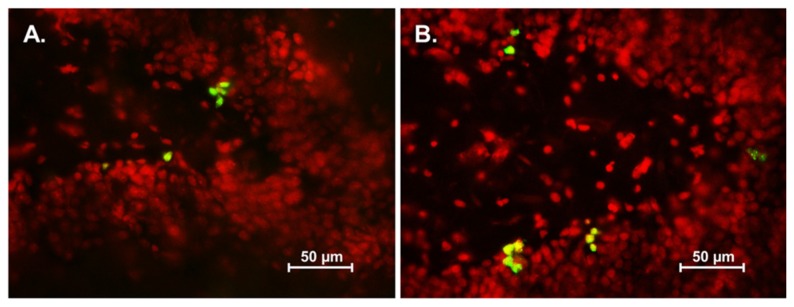

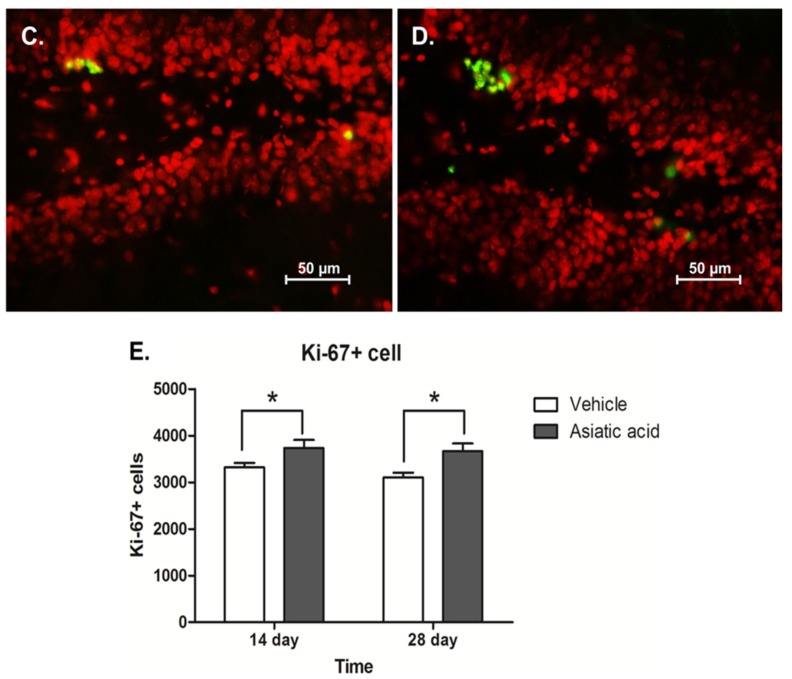
The number of Ki-67 positive cells in the SGZ (subgranular zone) of the hippocampal dentate gyrus. Ki-67 positive cells are stained green in the SGZ of the dentate gyrus (section counterstained with red nuclear dye, PI; (**A**) = control 14 day; (**B**) = asiatic acid 14 day; (**C**) = control 28 day and (**D**) = asiatic acid 28 day). The level of proliferating cells in animals receiving asiatic acid for both 14 and 28 days were significantly higher than controls (* *p* < 0.05) (**E**). A two-way Analysis of variance with Fisher's Least Significant Difference *post hoc* test was used to compare between all groups.

The level of cell proliferation in the SGZ was quantified using Ki-67 immunohistochemistry. Animals which had received asiatic acid for both 14 and 28 days showed significantly higher in the number of Ki-67 positive cells compared with controls (mean ± SEM; asiatic acid 14 days-treated group: 3733 ± 423.5 cells and control 14 days-treated group: 3319 ± 230.9 cells; asiatic acid 28 days-treated group: 3665 ± 407.1 cells and control 28 days-treated group: 3103 ± 256.6 cells; F (1,20) = 12.31; *n* = 6; *p* = 0.0022, two-way ANOVA, LSD *post-hoc* test, Figure 3E). These results indicate that asiatic acid increases cell proliferation in the SGZ of the hippocampal dentate gyrus.

### 3.3. Effect of Asiatic Acid on Hippocampal Notch1 and DCX

**Figure 4:**
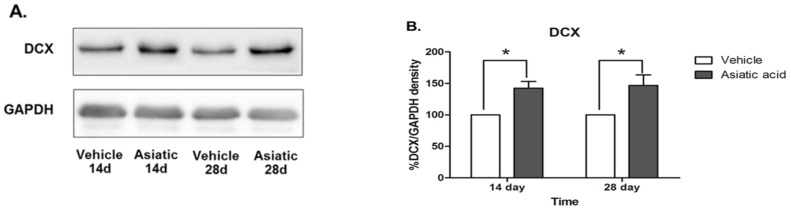

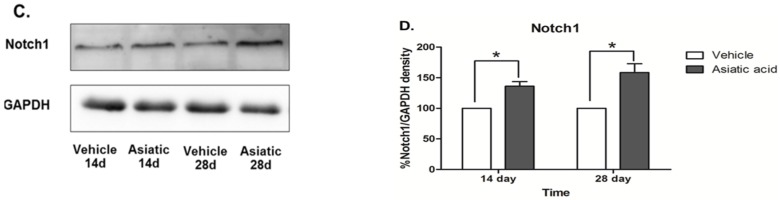
Photographs of DCX (Stem Cells and Doublecortin) and Notch1 protein expressions. DCX expression in the hippocampus of asiatic acid-treated animals showed a significant increase compared to the control animals in both time periods (**A**, **B**; * *p* < 0.05, ). Animals treated with asiatic acid for both time periods had significantly higher Notch1 expressions than controls (**C**, **D**; * *p* < 0.05). Levels of Notch1 and DCX were normalized to GAPDH (Glyceraldehyde 3-phosphate dehydrogenase). Two-way ANOVA with LSD *post hoc* test was used to compare between all groups.

Western blotting was done to determine the effect of asiatic acid on levels of Notch1 and DCX in the hippocampus. Asiatic acid produced an increase in DCX protein expression in animals that were administered asiatic acid for both 14 and 28 days (F (1, 24) = 20.07; *n* = 7; *p* < 0.001, two-way ANOVA, LSD *post-hoc* test, Figure 4 A,B). Notch1 protein expression was significantly increased in both time periods of asiatic acid-treated groups compared with controls (F (1, 23) = 39.62; *n* = 7; *p* < 0.001, two-way ANOVA, LSD *post-hoc* test, Figure 4 C,D). These results suggest that asiatic acid can increase the levels of a neural stem cell marker and the number of immature neurons in the SGZ of the dentate gyrus.

## 4. Discussion

Adult hippocampal neurogenesis and hippocampal-dependent learning can be influenced by various factors including physiological, pharmacological and environmental stimuli. Increases in hippocampal neurogenesis are associated with enhanced cognition as newly generated neurons are used in spatial navigation and pattern separation [29,30]. Other parts of the brain, including the prefrontal cortex and posterior pareitalcortex, are important to spatial memory [31]. However, many studies have shown that cell proliferation/neurogenesis in the hippocampal dentate gyrus plays a crucial in spatial memory using novel object location tests [18,19,27]. The NOL behavioral test was chosen as a test of spatial memory, as performance in this test is hippocampal-dependent [20]. It relies on the spontaneous preference of the animal and does not require positive or negative reinforcements. A previous study found that asiatic acid, a triterpenoid from *Centella asiatica*, can improve learning and memory in an animal model [32]. The present study shows that asiatic acid improves spatial memory. Animals in all groups were able to discriminate between two identical objects in novel and familiar locations and spent significantly more time on the objects in the novel locations. However, rats that had received asiatic acid for 28 days spent significantly more time attending the object in a novel location compared to control animals and animals which had received asiatic acid for 14 days. Thus, it appears that asiatic acid can enhance spatial memory after administration for 28 days but not after 14 days of treatment. Asiatic acid is likely to have an effect on spatial memory by regulating the *N*-methyl-d-aspartate (NMDA) receptor [8,33] or acethylcoline synthesis [32]. Therefore, further study is required to investigate the effect of asiatic acid on the NOL test by regulating the NMDA receptor or acethylcholine synthesis.

Spatial working memory is strongly correlated with the degree of neurogenesis in the hippocampal dentate gyrus with increased neurogenesis being associated with improved memory and reduced neurogenesis with memory deficits [34,35,36]. In the present study, the effect of asiatic acid on cell proliferation in the SGZ of the hippocampus was determined. The levels of proliferating cells in animals administered asiatic acid after 14 and 28 days were significantly higher than control animals. These results indicate that asiatic acid increases cell proliferation in the SGZ of dentate gyrus.

Asiatic acid has been shown to exhibit neuroprotective and antioxidant properties [6]. Oral administration of asiatic acid in animal models significantly restored lipid peroxidation, glutathione and activity of superoxide dismutase (SOD) in the hippocampus and cortex to control levels [33] along with an improvement of antioxidant activities [37]. Previous studies have reported that an increase in oxidant production and a reduction of the antioxidant capacity of the cell can decrease the survival and differentiation of mesencephalic precursors [38,39] and neural crest stem cells [40]. Therefore, the effect of asiatic acid on production of new hippocampal cells was examined in the present study.

DCX is a microtubule-associated protein that is expressed mainly in migrating neuronal precursors of the developing central nervous system [41]. DCX is expressed by immature neurons as they become integrated into the dentate gyrus and as such can be used as a measure of neurogenesis [42,43,44]. Asiatic acid treatment increased DCX protein levels within the hippocampus after both 14 and 28 days treatment, indicating that asiatic acid was stimulating neurogenesis possibly by increasing both cell proliferation and survival. Recently it has been demonstrated that DCX is expressed in non-neurogenic brain regions at levels below immunohistochemical detection [45]. It is likely that expression in these regions indicates ongoing changes in neuronal plasticity.

The Notch1 receptor is expressed on neural stem cells where it regulates cell differentiation and neuronal cell fate specifications. Moreover, Notch1 also plays an important role in neuronal differentiation, survival and plasticity and is also expressed in the dendrites of mature neurons [46,47,48,49]. Recent studies have reported that a reduction in Notch1 expression is associated with a decrease of neurogenesis in the hippocampal dentate gyrus and spatial memory deficits [20,46,47,50,51]. The present study showed increased levels of Notch 1 after 14 and 28 days of asiatic acid treatment. This change correlates with the increase in cell proliferation and DCX expression and is a further indication that asiatic acid treatment increases hippocampal neurogenesis. In conclusion, the present findings indicate that administration of asiatic acid is effective in enhancing cell proliferation in the SGZ of the hippocampus and spatial working memory. Hence, asiatic acid might be useful in increasing learning and memory in situations of cognitive decline. Further research on other mechanisms of asiatic acid on neurogenesis will help achieve the understanding required to aid in prevention and resolution of memory deficits in patients.

## References

[1] Zheng C.J., Qin L.P. (2007). Chemical components of centella asiatica and their bioactivities. Zhong Xi Yi Jie He Xue Bao.

[2] Nasir M.N., Abdullah J., Habsah M., Ghani R.I., Rammes G. (2012). Inhibitory effect of asiatic acid on acetylcholinesterase, excitatory post synaptic potential and locomotor activity. Phytomedicine.

[3] Nasir M.N., Habsah M., Zamzuri I., Rammes G., Hasnan J., Abdullah J. (2011). Effects of asiatic acid on passive and active avoidance task in male spraque-dawley rats. J. Ethnopharmacol..

[4] Zhang X., Wu J., Dou Y., Xia B., Rong W., Rimbach G., Lou Y. (2012). Asiatic acid protects primary neurons against C 2-ceramide-induced apoptosis. Eur. J. Pharmacol..

[5] Orhan I.E. (2012). *Centella asiatica* (L.) Urban: From traditional medicine to modern medicine with neuroprotective potential. Evid. Based Complement. Altern. Med..

[6] Lee M.K., Kim S.R., Sung S.H., Lim D., Kim H., Choi H., Park H.K., Je S., Ki Y.C. (2000). Asiatic acid derivatives protect cultured cortical neurons from glutamate-induced excitotoxicity. Res. Commun. Mol. Pathol. Pharmacol..

[7] Krishnamurthy R.G., Senut M.C., Zemke D., Min J., Frenkel M.B., Greenberg E.J., Yu S.W., Ahn N., Goudreau J., Kassab M. (2009). Asiatic acid, a pentacyclic triterpene from centella asiatica, is neuroprotective in a mouse model of focal cerebral ischemia. J. Neurosci. Res..

[8] Lee K.Y., Bae O.N., Weinstock S., Kassab M., Majid A. (2014). Neuroprotective effect of asiatic acid in rat model of focal embolic stroke. Biol. Pharm. Bull..

[9] Ming G.L., Song H. (2005). Adult neurogenesis in the mammalian central nervous system. Annu. Rev. Neurosci..

[10] Taupin P. (2005). Adult neurogenesis in the mammalian central nervous system: Functionality and potential clinical interest. Med. Sci. Monit..

[11] Curtis M.A., Kam M., Nannmark U., Anderson M.F., Axell M.Z., Wikkelso C., Holtas S., van Roon-Mom W.M., Bjork-Eriksson T., Nordborg C. (2007). Human neuroblasts migrate to the olfactory bulb via a lateral ventricular extension. Science.

[12] Toni N., Laplagne D.A., Zhao C., Lombardi G., Ribak C.E., Gage F.H., Schinder A.F. (2008). Neurons born in the adult dentate gyrus form functional synapses with target cells. Nat. Neurosci..

[13] Kitabatake Y., Sailor K.A., Ming G.L., Song H. (2007). Adult neurogenesis and hippocampal memory function: New cells, more plasticity, new memories?. Neurosurg. Clin. N. Am..

[14] Jenkins T.A., Amin E., Pearce J.M., Brown M.W., Aggleton J.P. (2004). Novel spatial arrangements of familiar visual stimuli promote activity in the rat hippocampal formation but not the parahippocampal cortices: A c-Fos expression study. Neuroscience.

[15] Goodrich-Hunsaker N.J., Hunsaker M.R., Kesner R.P. (2008). The interactions and dissociations of the dorsal hippocampus subregions: How the dentate gyrus, CA3, and CA1 process spatial information. Behav. Neurosci..

[16] Kee N., Teixeira C.M., Wang A.H., Frankland P.W. (2007). Preferential incorporation of adult-generated granule cells into spatial memory networks in the dentate gyrus. Nat. Neurosci..

[17] Lyons L., ElBeltagy M., Umka J., Markwick R., Startin C., Bennett G., Wigmore P. (2011). Fluoxetine reverses the memory impairment and reduction in proliferation and survival of hippocampal cells caused by methotrexate chemotherapy. Psychopharmacology.

[18] ELBeltagy M., Mustafa S., Umka J., Lyons L., Salman A., Dormon K., Allcock C., Bennett G., Wigmore P. (2012). The effect of 5-fluorouracil on the long term survival and proliferation of cells in the rat hippocampus. Brain Res. Bull..

[19] Mustafa S., Walker A., Bennett G., Wigmore P.M. (2008). 5-fluorouracil chemotherapy affects spatial working memory and newborn neurons in the adult rat hippocampus. Eur. J. Neurosci..

[20] Umka J., Mustafa S., ElBeltagy M., Thorpe A., Latif L., Bennett G., Wigmore P.M. (2010). Valproic acid reduces spatial working memory and cell proliferation in the hippocampus. Neuroscience.

[21] Encinas J.M., Vaahtokari A., Enikolopov G. (2006). Fluoxetine targets early progenitor cells in the adult brain. Proc. Natl. Acad. Sci. USA.

[22] Scholzen T., Gerdes J. (2000). The Ki-67 protein: From the known and the unknown. J. Cell. Physiol..

[23] Dix S.L., Aggleton J.P. (1999). Extending the spontaneous preference test of recognition: Evidence of object-location and object-context recognition. Behav. Brain Res..

[24] King M.V., Sleight A.J., Woolley M.L., Topham I.A., Marsden C.A., Fone K.C.F. (2004). 5-HT 6 receptor antagonists reverse delay-dependent deficits in novel object discrimination by enhancing consolidation—An effect sensitive to nmda receptor antagonism. Neuropharmacology.

[25] Weible A.P., Rowland D.C., Pang R., Kentros C. (2009). Neural correlates of novel object and novel location recognition behavior in the mouse anterior cingulate cortex. J. Neurophysiol..

[26] Mayhew T.M., Burton G.J. (1988). Methodological problems in placental morphometry: Apologia for the use of stereology based on sound sampling practice. Placenta.

[27] ELBeltagy M., Mustafa S., Umka J., Lyons L., Salman A., Chur-Yoe G.T., Bhalla N., Bennett G., Wigmore P.M. (2010). Fluoxetine improves the memory deficits caused by the chemotherapy agent 5-fluorouracil. Behav. Brain Res..

[28] Huang G.J., Herbert J. (2006). Stimulation of neurogenesis in the hippocampus of the adult rat by fluoxetine requires rhythmic change in corticosterone. Biol. Psychiatry.

[29] Gould E., Beylin A., Tanapat P., Reeves A., Shors T.J. (1999). Learning enhances adult neurogenesis in the hippocampal formation. Nat. Neurosci..

[30] Trouche S., Bontempi B., Roullet P., Rampon C. (2009). Recruitment of adult-generated neurons into functional hippocampal networks contributes to updating and strengthening of spatial memory. Proc. Natl. Acad. Sci. USA.

[31] Van Asselen M., Kessels R.P.C., Neggers S.F.W., Kappelle L.J., Frijns C.J.M., Postma A. (2006). Brain areas involved in spatial working memory. Neuropsychologia.

[32] Kim S.R., Koo K.A., Lee M.K., Park H.G., Jew S.S., Cha K.H., Kim Y.C. (2004). Asiatic acid derivatives enhance cognitive performance partly by improving acetylcholine synthesis. J. Pharm. Pharmacol..

[33] Xu M.F., Xiong Y.Y., Liu J.K., Qian J.J., Zhu L., Gao J. (2012). Asiatic acid, a pentacyclic triterpene in centella asiatica, attenuates glutamate-induced cognitive deficits in mice and apoptosis in SH-SY5Y cells. Acta Pharmacol. Sin..

[34] Poirier G.L., Amin E., Aggleton J.P. (2008). Qualitatively different hippocampal subfield engagement emerges with mastery of a spatial memory task by rats. J. Neurosci..

[35] Zhao C., Deng W., Gage F.H. (2008). Mechanisms and functional implications of adult neurogenesis. Cell.

[36] Ehninger D., Kempermann G. (2008). Neurogenesis in the adult hippocampus. Cell Tissue Res..

[37] Ramachandran V., Saravanan R. (2013). Efficacy of asiatic acid, a pentacyclic triterpene on attenuating the key enzymes activities of carbohydrate metabolism in streptozotocin-induced diabetic rats. Phytomedicine.

[38] Lee J.Y., Koh H.C., Chang M.Y., Park C.H., Lee Y.S., Lee S.H. (2003). Erythropoietin and bone morphogenetic protein 7 mediate ascorbate-induced dopaminergic differentiation from embryonic mesencephalic precursors. Neuroreport.

[39] Studer L., Csete M., Lee S.H., Kabbani N., Walikonis J., Wold B., McKay R. (2000). Enhanced proliferation, survival, and dopaminergic differentiation of cns precursors in lowered oxygen. J. Neurosci..

[40] Morrison S.J., Csete M., Groves A.K., Melega W., Wold B., Anderson D.J. (2000). Culture in reduced levels of oxygen promotes clonogenic sympathoadrenal differentiation by isolated neural crest stem cells. J. Neurosci..

[41] Rao M.S., Shetty A.K. (2004). Efficacy of doublecortin as a marker to analyse the absolute number and dendritic growth of newly generated neurons in the adult dentate gyrus. Eur. J. Neurosci..

[42] Couillard-Despres S., Winner B., Schaubeck S., Aigner R., Vroemen M., Weidner N., Bogdahn U., Winkler J., Kuhn H.G., Aigner L. (2005). Doublecortin expression levels in adult brain reflect neurogenesis. Eur. J. Neurosci..

[43] Francis F., Koulakoff A., Boucher D., Chafey P., Schaar B., Vinet M.C., Friocourt G., McDonnell N., Reiner O., Kahn A. (1999). Doublecortin is a developmentally regulated, microtubule-associated protein expressed in migrating and differentiating neurons. Neuron.

[44] Gleeson J.G., Lin P.T., Flanagan L.A., Walsh C.A. (1999). Doublecortin is a microtubule-associated protein and is expressed widely by migrating neurons. Neuron.

[45] Kremer T., Jagasia R., Herrmann A., Matile H., Borroni E., Francis F., Kuhn H.G., Czech C. (2013). Analysis of adult neurogenesis: Evidence for a prominent “non-neurogenic” dcx-protein pool in rodent brain. PLoS ONE.

[46] Alberi L., Liu S., Wang Y., Badie R., Smith-Hicks C., Wu J., Pierfelice T.J., Abazyan B., Mattson M.P., Kuhl D. (2011). Activity-induced notch signaling in neurons requires Arc/Arg3.1 and is essential for synaptic plasticity in hippocampal networks. Neuron.

[47] Wang Y., Chan S.L., Miele L., Yao P.J., Mackes J., Ingram D.K., Mattson M.P., Furukawa K. (2004). Involvement of notch signaling in hippocampal synaptic plasticity. Proc. Natl. Acad. Sci. USA.

[48] Breunig J.J., Silbereis J., Vaccarino F.M., Sestan N., Rakic P. (2007). Notch regulates cell fate and dendrite morphology of newborn neurons in the postnatal dentate gyrus. Proc. Natl. Acad. Sci. USA.

[49] Costa R.M., Honjo T., Silva A.J. (2003). Learning and memory deficits in notch mutant mice. Curr. Biol..

[50] Stump G., Durrer A., Klein A.L., Lutolf S., Suter U., Taylor V. (2002). Notch1 and its ligands delta-like and jagged are expressed and active in distinct cell populations in the postnatal mouse brain. Mech. Dev..

[51] Costa R.M., Drew C., Silva A.J. (2005). Notch to remember. Trends Neurosci..

